# Editorial: Effect of neurophysiological conditions and mental workload on physical and cognitive performances: a multidimensional perspective

**DOI:** 10.3389/fnrgo.2023.1215376

**Published:** 2023-05-17

**Authors:** David Perpetuini, Damiano Formenti, Jose Ignacio Priego-Quesada, Arcangelo Merla

**Affiliations:** ^1^Department of Neuroscience, Imaging and Clinical Sciences, University of Studies G. d'Annunzio University of Chieti and Pescara, Chieti, Italy; ^2^Department of Biotechnology and Life Sciences, University of Insubria, Varese, Italy; ^3^Department of Physical Education and Sports, Faculty of Physical Activity and Sport Sciences, Universitat de València, Valencia, Spain; ^4^Department of Engineering and Geology, G. d'Annunzio University of Chieti and Pescara, Pescara, Italy

**Keywords:** mental workload (MWL), physical performances, cognitive performances, neuromotor rehabilitation, wellbeing

The Research Topic “*Effect of Neurophysiological Conditions and Mental Workload on Physical and Cognitive Performances: a Multidimensional Perspective*” compiles recent advances in the multidimensional assessment of the effect of neurophysiological status and mental workload on physical and cognitive performance (Aghajani et al., 2017; Perpetuini et al., 2022). Due to technological and data analytics tool advancements, non-invasive and real-time monitoring of the activity of the human central and autonomic nervous systems is now possible, facilitating the quantification of the relationship between physiology and human performance (Ashkanasy, 2004; Cardone et al., 2022). Moreover, questionnaires, whose scores highly correlate with physiological responses (Noyes and Bruneau, 2007; Perpetuini et al., 2021), could be administered to assess the psychological conditions of individuals during the execution of various tasks.

Most of the studies published on this Research Topic have been focused on populations with special needs or pathologies. However, the first manuscript to be published was that of Rinella et al., in which the authors investigated the relationship between the digit ratio (D2:D4) and state and/or trait anxiety in healthy adults, and whether there are gender-specific differences. In this study, 125 participants of both sexes filled in the State-Trait Anxiety Inventory (STAI-Y) questionnaire and their D2:D4 ratio was calculated. According to the results, a low D2:D4 ratio (< 1) can be considered a protective factor against anxiety in both men and women, and this protection appears to be lifelong. In addition, a significant negative relationship was found between age, state, and trait anxiety, with no gender differences.

After this first study, the rest of the publications evaluated diseased pediatric populations (Rast and Labruyère), populations with immunodeficiency virus (Hardy and Hinkin), Down syndrome (Post et al.), spinal cord injury (Nhan et al.), stroke patients (Loria et al.), or Parkinson's Disease (PD) (Harro et al.).

In this sense, Rast and Labruyère discussed the use of sensor-based technology to monitor the motor activities of neuromotor impairments in infants and adolescents. The authors surveyed health professionals involved in pediatric rehabilitation for their opinions on the use of this technology. The results of the survey indicated that health professionals believe sensor-based outcomes can be beneficial for monitoring motor activities and delivering personalized interventions. However, the survey also revealed potential confounding variables that may influence the participants' responses. Overall, the paper discusses the prospective benefits and difficulties of using sensor-based technology in pediatric rehabilitation.

Hardy and Hinkin used the NASA-Task Load Index questionnaire (Noyes and Bruneau, 2007) to assess the workload of performing a computerized monitoring task of 32 adults positive for the human immunodeficiency virus. The study found that tracking performance decreased as task difficulty increased, while workload ratings tended to increase. The scores on the Mental Demand subscale revealed substantial individual differences in workload. Mental Demand was discovered to be associated with age and a diagnosis of acquired immune deficiency syndrome.

Post et al. aimed to investigate the effects of a 10-week resistance training program on motor behavior, cognitive function, mood, and physical fitness in young adults with Down syndrome. The results showed significant improvements in locomotor and object control skills, cognitive performance, mood, physical strength, endurance, and lean body mass. The participants also reported increased self-confidence, ability to complete daily activities, and interest in exercise, physical activity, nutrition, and overall health. The study suggests that a properly designed resistance training program can improve the health status and quality of life of people with Down syndrome.

The study by Nhan et al. examined the effect of acute submaximal exercise on cognition and brain-derived neurotrophic factor (BDNF) in spinal cord injury patients. On separate days, eight individuals with traumatic spinal cord injury performed submaximal intensity arm cycling or time-matched quiet rest. Serum and plasma levels of blood-borne BDNF were measured, and cognition was evaluated using the Stroop Test and Task-Switching Test. The results demonstrated that acute exercise based on guidelines did not increase BDNF or enhance aspects of cognition in spinal cord injury patients. This study lays the groundwork for future research on exercise as a therapeutic strategy for promoting mental health in spinal cord injury patients.

Loria et al. examined the use of accelerometers to evaluate the function of the paretic limb during therapeutic exercises in stroke patients. The patients participated in an auditory-motor intervention in which reaching movements of the paralyzed limb were mapped onto digital musical instruments and sound tablets. To quantify the volitional control and temporal consistency of the paretic limb movements, the resulting acceleration profiles were analyzed. Significant improvements in the acceleration of the paretic limb correlated with improvements in clinical assessments of motor function. These results indicate that accelerometry-based measures may be beneficial in stroke rehabilitation.

The research of Harro et al. investigated the effects of Nordic Walking exercise on walking function, motor/non-motor PD symptoms, and serum BDNF in individuals with idiopathic PD. Twelve participants with mild to moderate idiopathic PD participated in 6 weeks of supervised Nordic Walking exercise training with individualized instruction, followed by 14 weeks of independent Nordic Walking exercise with remote guidance. Results demonstrated that Nordic Walking exercise enhanced walking endurance, daily step count, motor/non-motor PD symptoms, and serum BDNF levels, and interestingly, after 3 months of independent NW exercise, these benefits were maintained.

We believe that the articles on this Research Topic provide a fascinating summary of the current state of research on the effect of neurophysiological status and mental workload on human physical and cognitive performance. We hope that they will inspire additional research on this topic that should be not only scientifically rigorous but also highly applicable to real-world situations. In fact, monitoring physiological functions in healthy and pathological patients could pave the way for innovative evidence-based solutions in clinical practice and sports science to enhance the efficacy of training processes, clinical treatments, and human well-being in general.

## Author contributions

DP and DF wrote the original draft of this editorial. DP, DF, JP-Q, and AM reviewed and edited it. All the authors have approved the submitted version of this editorial.
